# Organotypic brain explant culture as a drug evaluation system for malignant brain tumors

**DOI:** 10.1002/cam4.1174

**Published:** 2017-10-04

**Authors:** Noriaki Minami, Yusuke Maeda, Shunsuke Shibao, Yoshimi Arima, Fumiharu Ohka, Yutaka Kondo, Koji Maruyama, Masatoshi Kusuhara, Takashi Sasayama, Eiji Kohmura, Hideyuki Saya, Oltea Sampetrean

**Affiliations:** ^1^ Division of Gene Regulation Institute for Advanced Medical Research Keio University School of Medicine Tokyo Japan; ^2^ Department of Neurosurgery Kobe University Graduate School of Medicine Kobe Hyogo Japan; ^3^ Department of Neurosurgery Nagoya University School of Medicine Nagoya Japan; ^4^ Division of Cancer Biology Nagoya University Graduate School of Medicine Nagoya Japan; ^5^ Experimental Animal Facility Shizuoka Cancer Center Research Institute Sunto‐gun Shizuoka Japan; ^6^ Regional Resources Division Shizuoka Cancer Center Research Institute Sunto‐gun Shizuoka Japan

**Keywords:** Drug screening, glioma stem cells, invasion, malignant brain tumors, organotypic slice culture

## Abstract

Therapeutic options for malignant brain tumors are limited, with new drugs being continuously evaluated. Organotypic brain slice culture has been adopted for neuroscience studies as a system that preserves brain architecture, cellular function, and the vascular network. However, the suitability of brain explants for anticancer drug evaluation has been unclear. We here adopted a mouse model of malignant glioma based on expression of H‐Ras^V12^ in *Ink4a/Arf* ^−/−^ neural stem/progenitor cells to establish tumor‐bearing brain explants from adult mice. We treated the slices with cisplatin, temozolomide, paclitaxel, or tranilast and investigated the minimal assays required to assess drug effects. Serial fluorescence‐based tumor imaging was sufficient for evaluation of cisplatin, a drug with a pronounced cytotoxic action, whereas immunostaining of cleaved caspase 3 (a marker of apoptosis) and of Ki67 (a marker of cell proliferation) was necessary for the assessment of temozolomide action and immunostaining for phosphorylated histone H3 (a marker of mitosis) allowed visualization of paclitaxel‐specific effects. Staining for cleaved caspase 3 was also informative in the assessment of drug toxicity for normal brain tissue. Incubation of explants with fluorescently labeled antibodies to CD31 allowed real‐time imaging of the microvascular network and complemented time‐lapse imaging of tumor cell invasion into surrounding tissue. Our results suggest that a combination of fluorescence imaging and immunohistological staining allows a unified assessment of the effects of various classes of drug on the survival, proliferation, and invasion of glioma cells, and that organotypic brain slice culture is therefore a useful tool for evaluation of antiglioma drugs.

## Introduction

Knowledge of the genetic and molecular underpinnings of malignant glioma has increased greatly in recent years 1, 2, 3. However, the development of new and effective drugs for this condition has not kept up with such advances 4. A lack of assays that balance feasibility, sufficient throughput, and reconstruction of the architecture and environment of the normal brain is one technical reason for the slow pace of drug discovery. For cancer in general, cell‐based assays 5 remain the gold standard for high‐throughput screening, whereas animal models are important for validation of promising lead compounds and evaluation of safety and dosing schedules. The gap between these two phases of drug development—namely, evaluation of the effects of agents identified by screening in systems that are multidimensional, encompassing a complex microenvironment and cellular heterogeneity—is being narrowed by the use of organoid and organotypic cultures 6, 7.

Although brain and brain tumor organoids have also been established 8, 9, organotypic or ex vivo culture of brain explants has been extensively adopted for studies of the physiology and development of the central nervous system. Such culture systems maintain organ and cellular architecture 10, while also preserving the integrity of the tumor–stroma interaction. They allow component cells to proliferate and migrate as well as support the function of specialized cells such as neurons 10, 11, 12, 13. Furthermore, in combination with confocal or multiphoton imaging, they provide single‐cell resolution for real‐time tracking studies 14, 15, 16. Such features have also made these systems an attractive model for cancer studies. However, in the case of glioma, organotypic slices have been adopted mostly for the evaluation of specific processes, such as tumor cell migration 15, 17, 18, tumor–stroma interaction 19, uptake of fluorescently labeled proteins 20, and metabolic exchange 21. Although brain explants have also been applied to examine the effects of specific compounds 15, a comprehensive assessment of such systems as a tool for evaluation of potential antiglioma drugs has not been performed to date.

We have now examined what type of investigations need to be performed with brain explants from tumor‐bearing mice in order to evaluate the effects of candidate drugs and what are the advantages of this approach. We found that a combination of real‐time imaging, tumor size quantification, and immunohistological staining is necessary for correct assessment of the effects of different classes of compound. The main advantage of this approach is that it allows the simultaneous appraisal of tumor growth, toxicity to surrounding brain tissue, and tumor cell invasion within the same experimental setting, thus providing a more precise and unified evaluation of the main end points for antiglioma therapies.

## Materials and Methods

### Mouse models, cell culture, and reagents

*Ink4a/Arf*–null neural stem/progenitor cells transduced with a vector for the oncoprotein H‐Ras^V12^ and the fluorescent protein dsRed (designated RasR cells) were established as described previously 22. The mosaic analysis with double markers (MADM) mouse model of glioma was also established as described previously 23, 24. The cell line PNMG106 was established from TP53 and NF1 double‐KO, GFP‐expressing cells isolated from a tumor formed in a *Tp53* ^+/−^;*Nf1* ^+/fl^ MADM mouse. All cells were maintained in culture medium conditioned to support neurosphere growth (neural stem medium, or NSM), consisting of serum‐free DMEM‐F12 supplemented with recombinant human epidermal growth factor and basic fibroblast growth factor, each at 20 ng/mL (PeproTech, Rocky Hill, NJ), as well as heparan sulfate at 200 ng/mL (Sigma‐Aldrich, St. Louis, MO) and B27 supplement without vitamin A (Invitrogen, Carlsbad, CA). Temozolomide was obtained from LKT Labs (St. Paul, MN), cisplatin from Nichi‐iko (Toyama, Japan), paclitaxel and tranilast from Sigma‐Aldrich, and recombinant human transforming growth factor‐*β*1 (TGF‐*β*1) from PeproTech.

### Orthotopic implantation

All animal experiments were approved by the Animal Care and Use Committee of Keio University School of Medicine. Orthotopic implantation of cells was performed as described previously 18. In brief, female C57BL/6J mice were anesthetized and placed into a stereotactic apparatus (David Kopf Instruments, Tujunga, CA) and either 5 × 10^4^ viable RasR cells or 1 × 10^5^ viable PNMG106 cells were injected into the right hemisphere 2.0 mm lateral to the bregma and 3 mm below the surface of the brain. Animals were monitored daily for the development of neurological deficits.

### Cell survival assay

Cell survival was assayed with the use of a WST‐8 assay kit (Dojindo Laboratories, Kumamoto, Japan). Cells were plated in standard 96‐well culture plates at a density of 5 × 10^4^ cells per well and were incubated for 24 h with or without the indicated drugs. Absorbance at 450 nm was then measured with a microplate reader (Perkin Elmer, Waltham, MA).

### Cell cycle analysis

Single‐cell suspensions were prepared in PBS, fixed overnight in 70% ethanol, and stained with propidium iodide (25 *μ*g/mL) for flow cytometric analysis with an Attune instrument (Thermo Fisher Scientific, Waltham, MA) and FlowJo software (Tree Star, San Carlos, CA).

### Sphere growth assay

Cells were plated in low‐binding 96‐well plates (Corning, Corning, NY) at a density of 1000 cells per well, with or without the indicated drugs. Images were acquired with a Biorevo BZ9000 inverted microscope (Keyence, Osaka, Japan) at 7 days after plating. Sphere area was quantified with the use of ImageJ software (NIH, Boston, MA). Mask images were generated for each sphere and a common threshold adjustment was applied to all resulting images before measurement of sphere area relative to that of the control group.

### Brain slice explants, drug treatment, and imaging

Brains were removed and briefly placed in ice‐cold DMEM‐F12 containing 1% penicillin–streptomycin mix (Nacalai Tesque, Kyoto, Japan). The tissue was then cut into 200‐*μ*m coronal slices with a LeicaVS1200 vibratome (Leica, Wetzlar, Germany). Explants were cultured on Millicell‐CM culture inserts (Merck Millipore, Billerica, MA) in glass‐bottom plates. Slices were maintained in NSM at 37°C under a humidified atmosphere of 5% CO_2_. The medium was changed every day. Images were acquired with an LV10i inverted confocal microscope (Olympus, Tokyo, Japan). Relative tumor area was measured with the use of ImageJ. Background correction was performed on the basis of the rolling ball algorithm, with the radius of the rolling ball set at 50 pixels. A common threshold adjustment was applied to all composite RGB images in order to select the tumor mass and scattered tumor foci. Selected tumor areas were measured, and the change in tumor size was expressed as: tumor size (day 4) minus tumor size (day 0).

### Immunohistochemical and immunohistofluorescence staining of brain slices

For immunohistological analysis, explants attached to Millicell‐CM culture plate inserts were fixed overnight with 4% paraformaldehyde, embedded in paraffin, and sectioned at a thickness of 3 *μ*m. Sections were stained with rabbit polyclonal antibodies to cleaved caspase 3 (Cell Signaling Technology, Danvers, MA), to Ki67 (clone SP‐6, Thermo Fisher Scientific), and to *γ*H2AX (phospho‐S139; Abcam, Cambridge, UK) or with a mouse monoclonal antibody to phosphorylated histone H3 (Cell Signaling Technology). Immune complexes were detected as described previously 18. Quantifications were performed with ImageJ.

### Invasion analysis

Tumor‐bearing brain explants were cultured for 24 h to achieve stabilization of the tissue. After the addition of TGF‐*β*1, tranilast, or DMSO vehicle at the indicated concentrations, time‐lapse imaging was performed with the use of an FV10i confocal microscope (Olympus). A *z*‐stack of 50 *μ*m was acquired every 30 min. The distance migrated by 30 randomly selected tumor cells within 2 h was measured for each treatment and the migration speed compared among groups. Initial and final fluorescence images were extracted with ImageJ software. The number of tumor cells or foci was quantified by counting the total number of discrete fluorescence signals with ImageJ.

### Visualization of blood vessels in organotypic explants

Mice were subjected to deep anesthesia, and 200 *μ*g of FITC‐conjugated isolectin B4 (Vector Laboratories Burlingame, CA, USA) were injected intracardially. The animals were then killed, and brain explants were established and incubated on cell culture inserts for at least 1 h. Alternatively, explants were incubated for 10 min with CD16/32 (1:300 dilution; BioLegend, San Diego, CA) to block IgG Fc receptors II/III, washed with PBS, and stained with phycoerythrin (PE)‐ or FITC‐conjugated antibodies to mouse CD31 (1:300, BioLegend) or to mouse Sca1 (1:300, BioLegend). Images were acquired with an FV10i confocal microscope (Olympus).

### Statistical analysis

Three to five independent replicates were performed for all experiments unless indicated otherwise. Quantitative data are presented as means ± SD from representative experiments. Statistical analysis was performed by Student's *t* test for comparisons between two groups or by one‐way ANOVA followed by the Tukey's posttest for comparison of multiple groups, with the use of JMP7 software (SAS Institute, Cary, NC) or GraphPad Prism (La Jolla, CA). A *P* < 0.05 was considered statistically significant.

## Results

### Optimization of explant culture for comprehensive analysis

**Figure 1:**
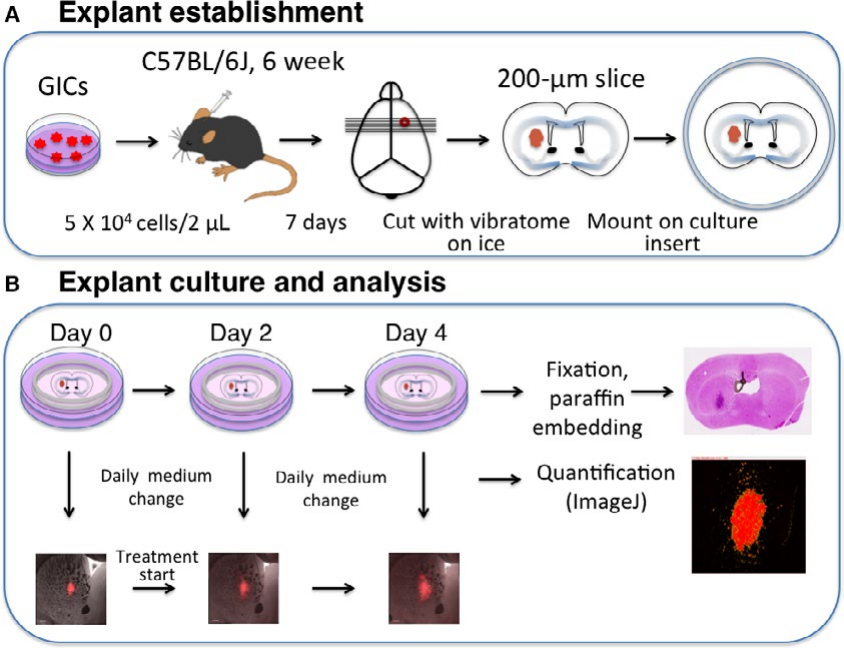
Explant establishment, culture, and analysis. (A) Explant establishment. dsRed‐expressing GICs (5 × 10^4^ RasR cells) are stereotactically implanted into the forebrain of C57BL/6J mice. Seven days after the injection, the tumor‐bearing brain is isolated and sliced at a thickness of 200 *μ*m with a vibrating‐blade microtome. The slices are then cultured at the air–fluid interface on cell culture inserts. (B) Explant culture and analysis. Explants are incubated in NSM supplemented with drugs or corresponding vehicle. The medium is replenished daily, and tumor cells are visualized every second day (days 0, 2, and 4). Tumor area is quantified with the use of ImageJ software. Immediately after the last imaging, explants are fixed overnight with 4% paraformaldehyde, embedded in paraffin, and sectioned at a thickness of 3 *μ*m for immunohistological analysis.

To take full advantage of the microenvironment provided by brain explants, we chose a syngeneic murine glioma model based on transduction of *Ink4a/Arf*–null neural stem/progenitor cells with the oncogene *H‐Ras* ^V12^. The transduced cells retain stem cell‐like properties and generate tumors that recapitulate the heterogeneity and histological features of human glioblastoma, thus acting as glioma‐initiating cells (GICs). Furthermore, the simultaneous expression of the fluorescent reporters dsRed or GFP allows visualization of tumor cells at the individual level in brain explants 18. Implantation of a known number of GICs predictably and reproducibly results in the formation of tumors of the same size at 7 days after the orthotopic grafting. We made use of this feature to establish explants from tumors of similar size for all drug‐screening experiments (Fig. 1A). Cell number was optimized in preliminary investigations so as to allow the establishment of three or four slices from the same mouse, thereby eliminating interanimal variability for comparisons between control and drug‐treated explants. Slice thickness was also optimized with regard to viability and optical penetration. Orthotopic implantation of 5 × 10^4^ GICs by stereotactic procedures as described previously 18 yielded tumors of (822 ± 174.8) ×(1500 ± 368.9) *μ*m^2^ at 7 days after engrafting. The brain was then removed and sectioned into 200‐*μ*m coronal slices with the use of a vibratome. Slices were mounted on culture inserts and maintained in culture at the air–fluid interface (Fig. 1A). Drug treatment experiments were designed to allow serial imaging and preservation of the explants at the end of the culture period. Images were acquired on the day of explant preparation (day 0) as well as on days 2 and 4. To ensure slice viability, maximal tumor growth, and maximal drug effects, we replenished the vehicle‐ or drug‐containing medium daily (Fig. 1B).

### Effects of cisplatin on explant cultures

To determine the minimal assays required for correct assessment of drug effects in the explant system, we chose four types of drug with different pharmacological actions. We first evaluated cisplatin (CDDP), a platinum compound that induces DNA damage by binding to the N‐7 reactive center on purine residues and promoting the formation of DNA–protein and DNA intrastrand cross‐links 25. A 50% reduction in cell viability was previously shown to be achieved at CDDP concentrations of 1–10 *μ*mol/L in glioma cell lines 26.

**Figure 2:**
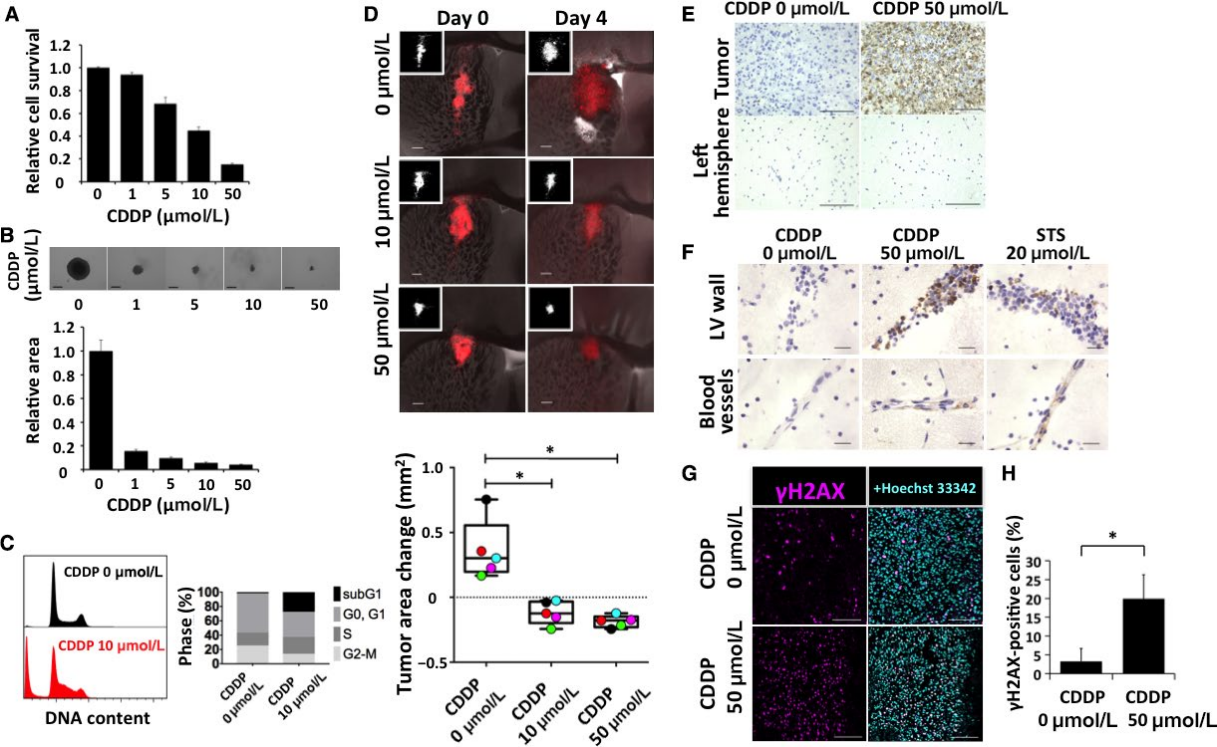
Effects of cisplatin (CDDP) on RasR cells. (A) Relative survival of RasR cells after exposure to the indicated concentrations of CDDP for 24 h. (B) Relative sphere growth after 7 days of drug treatment. Representative images of spheres are also shown. Scale bars, 300 *μ*m. (C) Flow cytometric analysis of cell cycle profile for RasR cells exposed to 0 or 10 *μ*mol/L CDDP for 48 h. (D) Overlay of red fluorescence and phase‐contrast images (as well as fluorescence images alone [insets]) for explants treated with the indicated concentrations of CDDP for 0 or 4 days. Scale bars, 300 *μ*m. The change in tumor area between day 0 and day 4 is also shown in a box‐and‐whisker plot and with individual values represented by colored circles. *P < 0.05. (E) Immunohistochemical staining of cleaved caspase 3 in tumor‐bearing explants from one experiment presented in (D), after fixation on day 4. Staining of the control left hemisphere is also shown. Scale bars, 100 *μ*m. (F) Immunohistochemical staining for cleaved caspase 3 around the lateral ventricle (LV) walls and blood vessels of tumor‐free explants treated with vehicle, 20 *μ*mol/L staurosporine (STS, positive control), or 50 *μ*mol/L CDDP. Scale bars, 20 *μ*m. (G) Immunohistofluorescence staining for *γ*H2AX and (H) quantification of the proportion of *γ*H2AX‐positive tumor cells after immunohistochemical staining of explants from (D). Scale bars, 100 *μ*m. *P < 0.05.

Treatment of our murine GICs (RasR cells) with CDDP for 24 h reduced cell survival in a concentration‐dependent manner (Fig. 2A). In a sphere‐formation assay, maximal inhibition of sphere growth by CDDP was apparent at a concentration of 10–50 *μ*mol/L (Fig. 2B). Cell cycle analysis revealed a marked increase in the proportion of the sub‐G_1_ population and an increase in the percentage of cells in S phase after treatment with 10 *μ*mol/L CDDP for 48 h (Fig. 2C).

Serial imaging of explants revealed that CDDP induced a significant reduction in tumor size at both 10 and 50 *μ*mol/L (Fig. 2D). This effect was evaluable by comparison of the fluorescent tumor area on confocal images and confirmed by quantification. Fixation of explants and immunohistochemical staining of cleaved caspase 3 at the end of the culture period confirmed a marked induction of tumor cell death by CDDP at 50 *μ*mol/L (Fig. 2E). However, damage to normal tissue was also apparent at this concentration, as evidenced by the presence of multiple cells positive for cleaved caspase 3 around the lateral ventricle walls and in normal blood vessels (Fig. 2F). Consistent with the results of previous in vitro experiments 26, immunohistofluorescence and immunohistochemical staining for *γ*H2AX revealed a significant increase in the proportion of cells positive for this marker of DNA double‐strand breaks in the CDDP‐treated tumors (Fig. 2G and H).

### Effects of temozolomide on explant cultures

We next evaluated the effects of temozolomide (TMZ), an alkylating agent of the imidazotetrazine class that induces methylation predominantly at the N‐7 or O‐6 positions of guanine residues and is a component of the standard treatment for malignant glioma 27, 28, 29. In contrast to CDDP, TMZ does not induce cross‐linking of DNA strands, suggestive of a milder action compared with that of CDDP 27.

**Figure 3:**
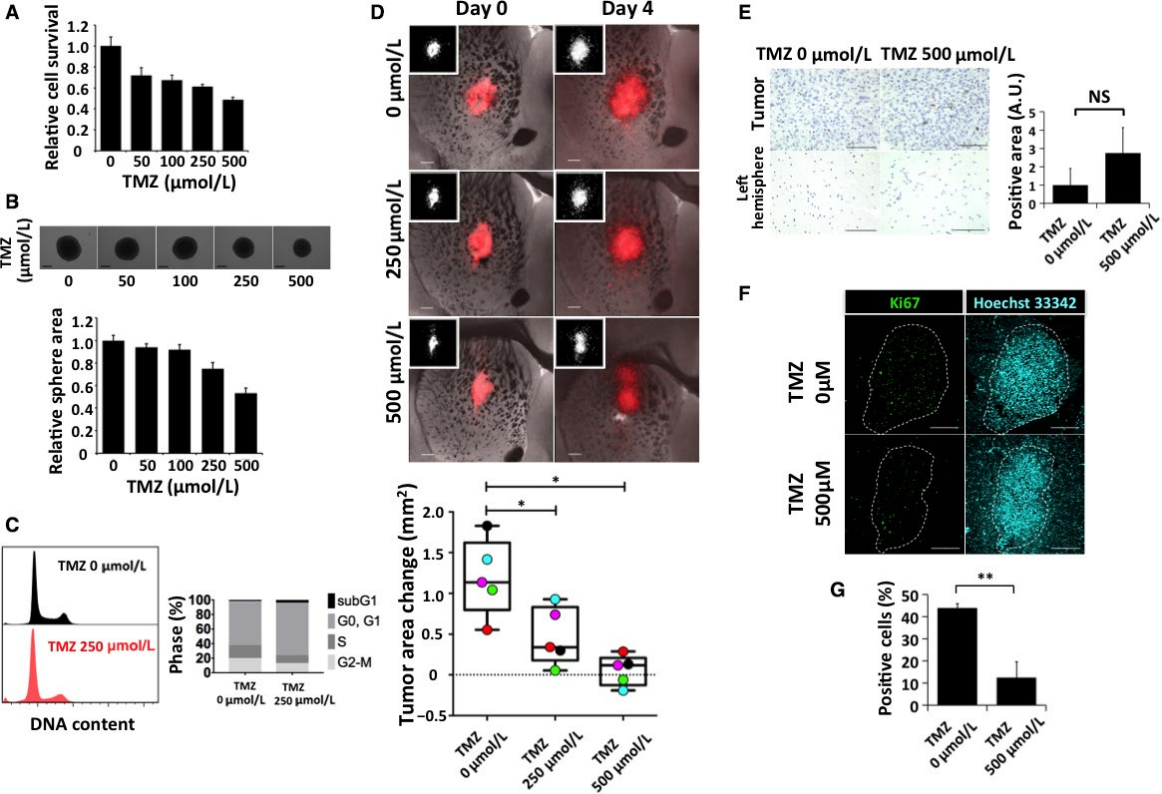
Effects of temozolomide (TMZ) on RasR cells. (A) Relative survival of RasR cells after exposure to the indicated concentrations of TMZ for 24 h. (B) Relative sphere growth after 7 days of drug treatment. Representative images of spheres are also shown. Scale bars, 300 *μ*m. (C) Flow cytometric analysis of cell cycle profile for RasR cells exposed to 0 or 250 *μ*mol/L TMZ for 48 h. (D) Overlay of red fluorescence and phase‐contrast images (as well as fluorescence images alone [insets]) for explants treated with the indicated concentrations of TMZ for 0 or 4 days. Scale bars, 300 *μ*m. The change in tumor area between day 0 and day 4 is also shown in a box‐and‐whisker plot and with individual values represented by colored circles. *P < 0.05. (E) Immunohistochemical staining for cleaved caspase 3 in the tumor‐bearing explants from (D) after fixation on day 4. Scale bars, 100 *μ*m. The tumor area positive for cleaved caspase 3 was determined in arbitrary units (A.U.). NS, not significant. (F) Immunohistofluorescence staining for Ki67 and (G) quantification of the proportion of Ki67‐positive tumor cells after immunohistochemical staining in explants from (D). Scale bars, 300 *μ*m. **P < 0.01.

We found that TMZ reduced the survival of our murine GICs, with a 51 ± 2.6% inhibitory effect apparent at a concentration of 500 *μ*mol/L (Fig. 3A). It also inhibited sphere growth by 47 ± 4.4% at this concentration (Fig. 3B). Cell cycle analysis showed that TMZ both induced cell death and attenuated cell proliferation, as revealed by a tendency to increase the size of the sub‐G_1_ and G_0_–G_1_ populations after 48 h treatment with either 250 *μ*mol/L or 500 *μ*mol/L TMZ (Figs. 3C and S1).

Serial imaging of explants showed that TMZ inhibited tumor growth in a concentration‐dependent manner (Fig. 3D). Tumor cell death was minimal, however, as revealed by the lack both of a mass reduction (Fig. 3D) and of a significant increase in cleaved caspase 3 positivity (Fig. 3E). In contrast, TMZ inhibited tumor cell proliferation as revealed by a significant decrease in positivity for the proliferation marker Ki67 (Fig. 3F and G).

### Effects of paclitaxel on explant cultures

The blood–brain barrier (BBB) limits the delivery of drugs to brain tumors, making it difficult to determine whether a lack of drug effectiveness is due to the absence of a pharmacological action or to such impeded delivery. In the explant system, compounds can directly enter the slice from the medium 11, with additional techniques being necessary to evaluate BBB penetration 30. We therefore next asked whether the explant system might be suitable for assessment of the pharmacological action of drugs that do not cross the BBB. Paclitaxel (PTX) binds to tubulin and thereby stabilizes microtubules, leading to aberrant mitosis and eventual cell death during mitosis or the subsequent G_1_ phase 31, 32.

**Figure 4:**
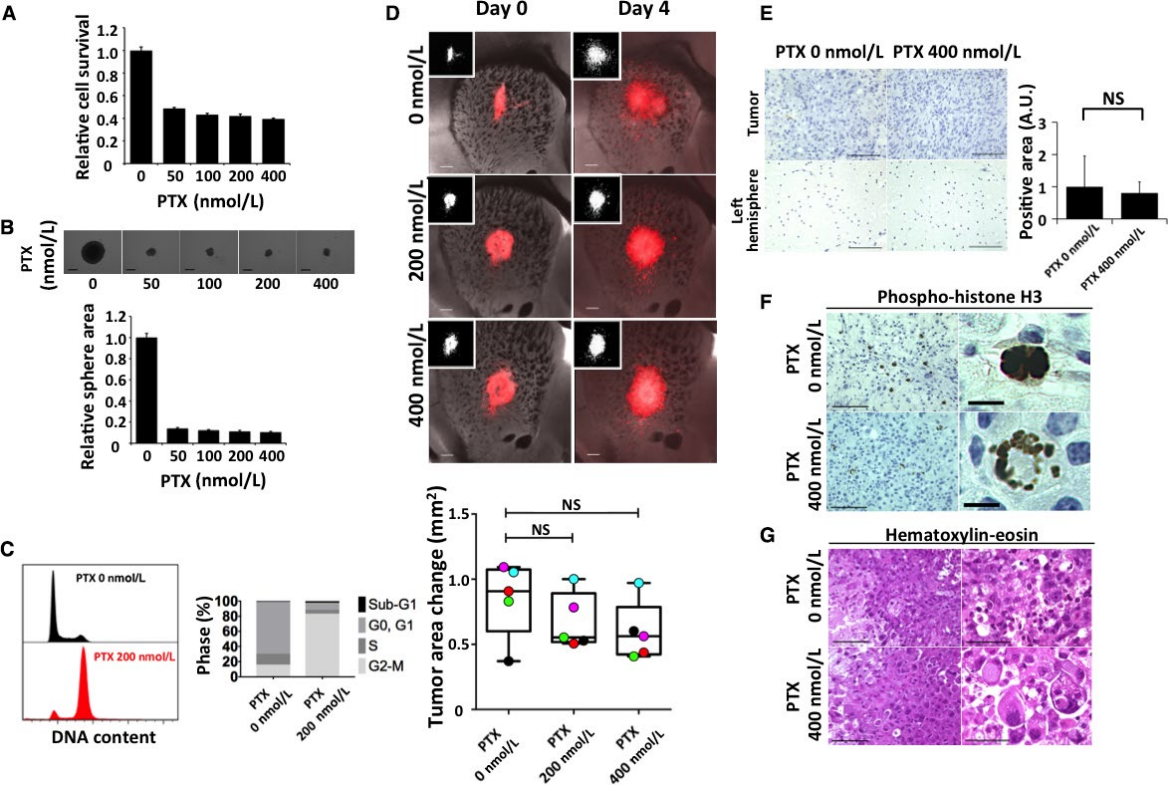
Effects of paclitaxel (PTX) on RasR cells. (A) Relative survival of RasR cells exposed to the indicated concentrations of PTX for 24 h. (B) Relative sphere growth after drug treatment for 7 days. Representative images of spheres are also shown. Scale bars, 300 *μ*m. (C) Flow cytometric analysis of cell cycle profile for RasR cells exposed to 0 or 200 nmol/L PTX for 15 h. (D) Overlay of red fluorescence and phase‐contrast images (or fluorescence images alone [insets]) for explants exposed to the indicated concentrations of PTX for 0 or 4 days. Scale bars, 300 *μ*m. The change in tumor area between day 0 and day 4 is also shown in a box‐and‐whisker plot with individual values represented by colored circles. Immunohistochemical staining for cleaved caspase 3 (E) or for phosphorylated histone H3 (F) as well as H&E staining (G) performed after fixation on day 4 for tumor‐bearing explants from (D). Scale bars: 100 *μ*m [(E) and left panels in (F) and (G)], 50 *μ*m [right panels in (G)], or 10 *μ*m [right panels in (F)].

The IC_50_ of PTX in glioma cell lines has previously been found to range from 1 to 10 nmol/L 33. In the case of our RasR cells, PTX concentrations of over 200 nmol/L induced maximal reduction in cell viability (Fig. 4A) and inhibition of sphere growth (Fig. 4B). Cell cycle analysis indicated a marked accumulation of cells in G_2_‐M (Fig. 4C). We examined the effects of 200 and 400 nmol/L PTX on explants. PTX had no apparent effect on tumor area, either at the qualitative or quantitative level (Fig. 4D). Consistent with these findings, PTX did not increase the proportion of tumor cells positive for cleaved caspase 3 (Fig. 4E). However, both immunohistochemical staining for the mitotic marker phosphorylated histone H3 (Fig. 4F) and H&E staining (Fig. 4G) revealed that PTX induced the formation of giant cells with abnormal nuclei and missegregated chromosomes, indicating that PTX had indeed acted on the tumor cells in the explant.

### Invasion analysis in explant culture

**Figure 5:**
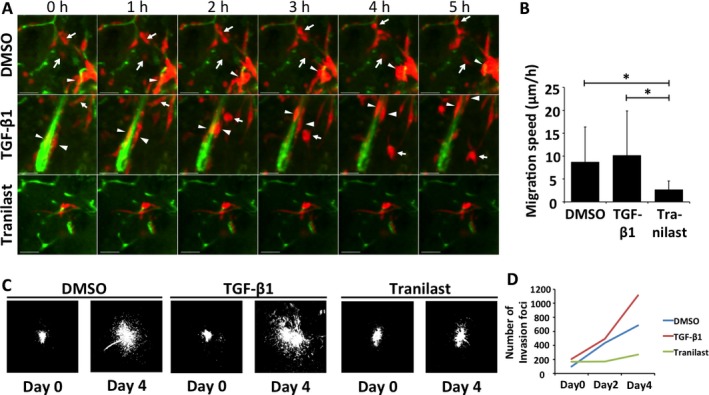
Invasion analysis of RasR cells ex vivo. (A) Sequential images showing the movement of RasR cells (red) along vascular structures visualized with FITC‐conjugated antibodies to Sca1 (green). Explants were treated with DMSO, TGF‐*β*1 (5 ng/mL), or tranilast (1 mmol/L) for the indicated times. The arrows and arrowheads indicate individual migrating tumor cells. Scale bars, 50 *μ*m. (B) Average speed of invading tumor cells determined as in (A). *P < 0.05. (C) Fluorescence signals of tumors at the beginning (day 0) and end (day 4) of explant treatment as in (A). (D) Average number of small invasion foci within the composite fields shown in (C).

Diffuse infiltration of tumor cells into the surrounding brain is a key issue in the treatment of malignant brain tumors 34, 35. We therefore asked whether our explant model is suitable for evaluation of anti‐invasion effects. Given that glioma cells often invade along blood vessels, we first attempted to visualize the vascular network. We found that the addition of PE‐conjugated antibodies specific for either the endothelial cell antigen CD31 or the stem cell antigen Sca1 36, 37 to the culture medium resulted in uptake specific for blood vessels, as shown by antibody colocalization with FITC‐conjugated isolectin B4 38 administered by intracardiac injection before establishment of the explants (Fig. S2). Given that neither TMZ‐ nor PTX‐treated explants manifested a reduction in the number of invasion foci (Figs. 3D, 4D), for evaluation of anti‐invasion effects we tested *N*‐[3,4‐dimethoxycinnamoyl]‐anthranilic acid (tranilast), an antiallergy drug that has been shown to inhibit the motility of glioma cells 39. Treatment of brain explants with 1 mmol/L tranilast reduced tumor cell motility compared with that apparent for explants exposed to DMSO vehicle, whereas TGF‐*β*1 slightly increased motility (Fig. 5) 40. These effects could be visualized by time‐lapse imaging of tumor cells and blood vessels (Fig. 5A) and could be quantified by calculating the speed of tumor cell invasion (Fig. 5B). After 4 days of treatment, the effects could be assessed by quantification of the number of invasion foci (Fig. 5C and D). Neither TGF‐*β*1 nor tranilast had a marked effect on the proportion of apoptotic or proliferating cells (Fig. S3). Both short‐ and medium‐term assays showed a tendency for tranilast to inhibit tumor cell invasion. Of note, this tendency was also apparent for murine glioma cells with a lower proliferative potential and different genetic background—namely, PNMG106 cells with homozygous deletion of *Tp53* and *Nf1* (Fig. S4 and Video S1).

## Discussion

Organotypic brain slice cultures preserve the cellular structure, vessel network, and extracellular matrix of the brain. We have here asked whether they are suitable for a systematic evaluation of the antitumor effects of various types of drug on murine glioma cells. We found that a combination of real‐time visualization of tumor cells with fixation and analysis of parameters such as proliferation indexes or mitotic indexes and markers of apoptosis offers a more complete and unified picture than performance of individual assays in vitro.

High‐grade gliomas are defined by marked cellular heterogeneity, which is in part due to the existence of stem‐like cells 41. These cells give rise to various progeny, ultimately forming a tumor that is a mix of cells that differ in proliferative ability and differentiation status. The presence of this functional hierarchy and the failure of screening based on conventional two‐dimensional cell culture to reproduce it accurately are thought to contribute to the failure in vivo of drugs that appear promising in vitro. Although most in vitro systems are devoid of the interactions among stem cells, nonstem tumor cells, and stromal cells, brain explants are able to sustain all three types of cells. For the present study, we therefore selected a murine model based on GICs with stem‐like properties 18.

Treatment of tumor‐bearing explants with CDDP provided two new insights into the use of organotypic brain slices for evaluation of anticancer drugs. First, it showed that, for drugs with a pronounced cytotoxic effect, sequential fluorescence imaging of the explants is sufficient for assessment of the antitumorigenic effect. Second, in contrast to cell culture‐based systems, the explant system simultaneously provided information regarding toxicity both to tumor cells and to the normal brain, as revealed by the accumulation of cleaved caspase 3‐positive cells in the ventricular walls and around blood vessels. Given that brain slices do not have a functional BBB, the toxicity to normal tissue in this system is presumably higher than it would be in vivo. However, opening of the BBB has been suggested as a means for a more efficient delivery of chemotherapeutic agents 42, and even osmotic modulators such as mannitol have been shown to transiently affect the BBB 43. Information on possible toxicities at near‐maximal delivery of a drug is therefore an important consideration for future clinical studies. Of note, low slice viability can lead to false positive results, while not all drugs may induce caspase‐dependent cell death. Therefore, evaluation of toxicity by multiple methods might be necessary. For live explants, uptake of propidium iodide (PI) and release of lactate dehydrogenase (LDH) can be used to detect changes in membrane permeability 10, while a TUNEL assay can complement assessment of cell death in fixed organotypic explants 7.

In the case of TMZ treatment, fluorescence imaging alone offered little insight into the effects of the drug. Immunohistological staining of the explants at the end of the experimental period revealed that, whereas the drug elicited little cell death, it induced marked growth arrest at the concentrations tested. Although these results are consistent with cell culture data, they further show that the antiproliferative effect of TMZ is also observed in the context of a tumor developing in a syngeneic microenvironment and are therefore suggestive of clinical efficacy. Furthermore, they indicate that at least basic molecular investigations are feasible in explants. Given that explants can also be subjected to protein extraction followed by immunoblot analysis or to cell isolation followed by flow cytometry, future studies might lead to the development of protocols for detailed mechanistic investigations.

Treatment with PTX confirmed the suitability of brain explants for evaluation of the effects of drugs that do not cross the BBB. The giant cells with aberrant nuclei and missegregated chromosomes detected by H&E staining and by immunohistochemical analysis of phosphorylated histone H3 are generated as a result of mitotic slippage and are indicative of PTX action 32. Their visualization in brain slices thus suggests that explants are suitable to obtain a proof of concept for the specificity of drug action. Furthermore, PTX‐treated slices, similar to TMZ‐treated ones, also highlighted a major issue in glioma treatment—that is, both drugs were unable to inhibit invasion of tumor cells into the surrounding brain.

Diffuse infiltration of tumor cells into the brain is thought to be one of the reasons that most antiglioma treatments ultimately fail 44. Furthermore, certain treatments such as antiangiogenic therapy with bevacizumab have been found to enhance such invasion 45. It is therefore important to evaluate the effects of target compounds not only on tumor cell survival but also on cell invasion. Brain explants allow simultaneous assessment of these two parameters, with the tumor cells residing in an environment that preserves the structure, density, stiffness, and extracellular matrix composition of brain tissue. Treatment of explants with tranilast revealed that time‐lapse images obtained by confocal microscopy, combined with quantification of invasion foci or migration speed, are informative with regard to the anti‐invasive potential of specific compounds. Furthermore, examination of the effects of tranilast in parallel with those of TGF‐*β*1 highlighted another important advantage of the brain explant system—namely, the ability to compare the effects of different drugs in slices from the same animal. This feature eliminates interanimal variability and reduces the number of animals required for such comparison studies.

Finally, we also showed that, by taking advantage of the direct access of fluorescently labeled antibodies to cell surface antigens such as CD31 or Sca1, we were able to readily and reproducibly visualize blood vessels in real time. This approach might also provide insight into the interactions of tumor cells with immune cells, as identified by cell surface antigens, or even into the behavior of CD133‐positive stem cells in xenograft slices. Of note, the interpretation of results regarding the interactions of tumor cells with blood vessels or immune cells will need to take into account the lack of blood flow and systemic modulation.

In addition to blood flow, organotypic slices cannot replicate the oxygen and drug gradient present in vivo. Since both oxygen and drugs penetrate the explants through the exposed surface and not from inside the blood vessels, this different dynamic also requires careful consideration.

In conclusion, our results indicate that a combination of fluorescence imaging and immunohistological staining allows a unified assessment of the effects of various classes of drug on the survival, proliferation, and invasion of tumor cells in brain explants, and they thus suggest that organotypic brain slices from tumor‐bearing mice are a useful tool for drug evaluation.

## Conflict of Interest

H. Saya has received commercial research grants from Daiichi Sankyo Co. Ltd., Eisai Co. Ltd., Nihon Noyaku Co. Ltd., Pola Pharma Inc., and AQUA Therapeutics Co. Ltd. All remaining authors declare no competing financial interests.

## Supporting information


**Figure S1.** Flow cytometric analysis of cell cycle profile for RasR cells exposed to 0 or to 500 μmol/L TMZ for 24 or 48 h (*n* = 1).
**Figure S2.** Confirmation of live staining of blood vessels in tumor‐free explants. Explants derived from mice injected intracardially (i.c.) with FITC‐conjugated isolectin B4 were subjected to blocking of Fc receptors followed by staining with PE‐conjugated antibodies to CD31 (top row) or to Sca1 (middle row). Alternatively, an explant derived from a noninjected mouse was stained with FITC‐conjugated antibodies to Sca1 and PE‐conjugated antibodies to CD31 after blocking of Fc receptors. Scale bars, 300 *µ*m.
**Figure S3.** Effects of tranilast and TGF‐β1 on apoptosis and cell proliferation in tumor‐bearing explants. Explants from (5C) were subjected to immunohistochemical staining for cleaved caspase 3 and Ki67 on day 4. Scale bars, 50 *µ*m.
**Figure S4.** Visualization of MADM mouse‐derived GFP‐positive Tp53^−^/^−^;Nf1^−^/^−^ glioma cells in organotypic slices prepared from recipient C57BL/6J mice at 13 days after implantation. Slices were treated with DMSO, TGF‐*β*1 (10 ng/mL), or tranilast (100 µmol/L) for 0 or 4 days. Scale bars, 100 *µ*m.
**Video S1.** Time‐lapse imaging of MADM mouse‐derived GFP‐positive Tp53^−^/^−^;Nf1^−^/^−^ glioma cells in organotypic slices prepared from recipient C57BL/6 mice. Slices were treated with DMSO, TGF‐*β*1 (10 ng/mL), or tranilast (100 *µ*mol/L). Images were acquired every 30 min.
