# Supplementary material for: Organotypic brain explant culture as a drug evaluation system for malignant brain tumors
Source: Cancer Med. 2017 Oct 4;6(11):2635–45. doi: 10.1002/cam4.1174 (PMC5673912; doi:10.1002/cam4.1174)
Supplement: Supplementary file 1 — Figure S1. Flow cytometric analysis of cell cycle profile for RasR cells exposed to 0 or to 500 μmol/L TMZ for 24 or 48 h (n = 1). Figure S2. Confirmation of live staining of blood vessels in tumor‐free explants. Explants derived from mice injected intracardially (i.c.) with FITC‐conjugated isolectin B4 were subjected to blocking of Fc receptors followed by staining with PE‐conjugated antibodies to CD31 (top row) or to Sca1 (middle row). Alternatively, an explant derived from a noninjected mouse was stained with FITC‐conjugated antibodies to Sca1 and PE‐conjugated antibodies to CD31 after blocking of Fc receptors. Scale bars, 300 µm. Figure S3. Effects of tranilast and TGF‐β1 on apoptosis and cell proliferation in tumor‐bearing explants. Explants from (5C) were subjected to immunohistochemical staining for cleaved caspase 3 and Ki67 on day 4. Scale bars, 50 µm. Figure S4. Visualization of MADM mouse‐derived GFP‐positive Tp53−/−;Nf1−/− glioma cells in organotypic slices prepared from recipient C57BL/6J mice at 13 days after implantation. Slices were treated with DMSO, TGF‐β1 (10 ng/mL), or tranilast (100 µmol/L) for 0 or 4 days. Scale bars, 100 µm. Video S1. Time‐lapse imaging of MADM mouse‐derived GFP‐positive Tp53−/−;Nf1−/− glioma cells in organotypic slices prepared from recipient C57BL/6 mice. Slices were treated with DMSO, TGF‐β1 (10 ng/mL), or tranilast (100 µmol/L). Images were acquired every 30 min. [file CAM4-6-2635-s001.pdf]

Supplemental material

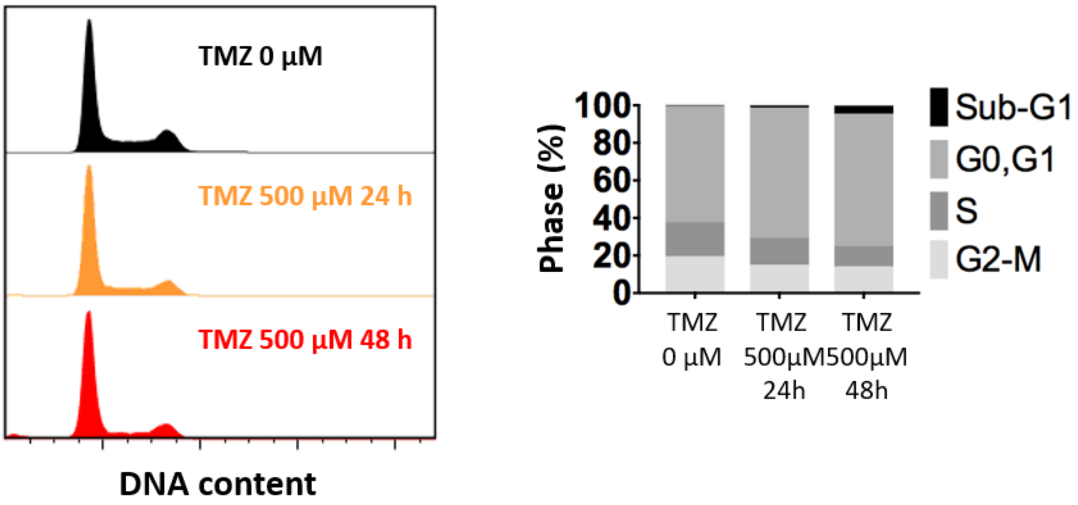

Fig. S1: Flow cytometric analysis of cell cycle profile for RasR cells exposed to 0 or to 500 µM TMZ for 24h or 48 h (n=1).

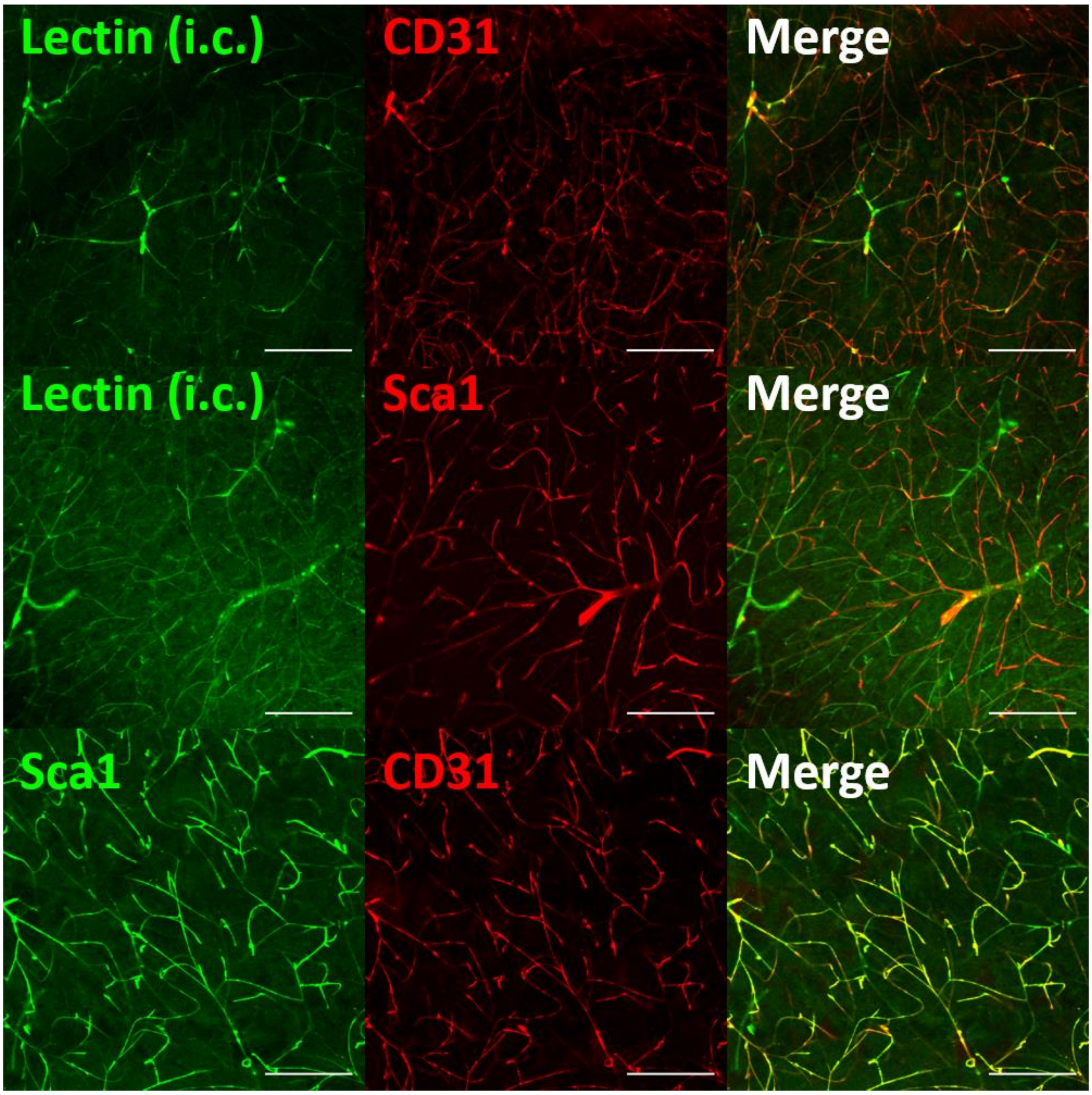

Fig. S2.: Confirmation of live staining of blood vessels in tumor-free explants. Explants derived from mice injected intracardially (i.c.) with FITC-conjugated isolectin B4 were subjected to blocking of Fc receptors followed by staining with PE-conjugated antibodies to CD31 (top row) or to Sca1 (middle row). Alternatively, an explant derived from a noninjected mouse was stained with FITC-conjugated antibodies to Sca1 and PE-conjugated antibodies to CD31 after blocking of Fc receptors. Scale bars, 300 μm.

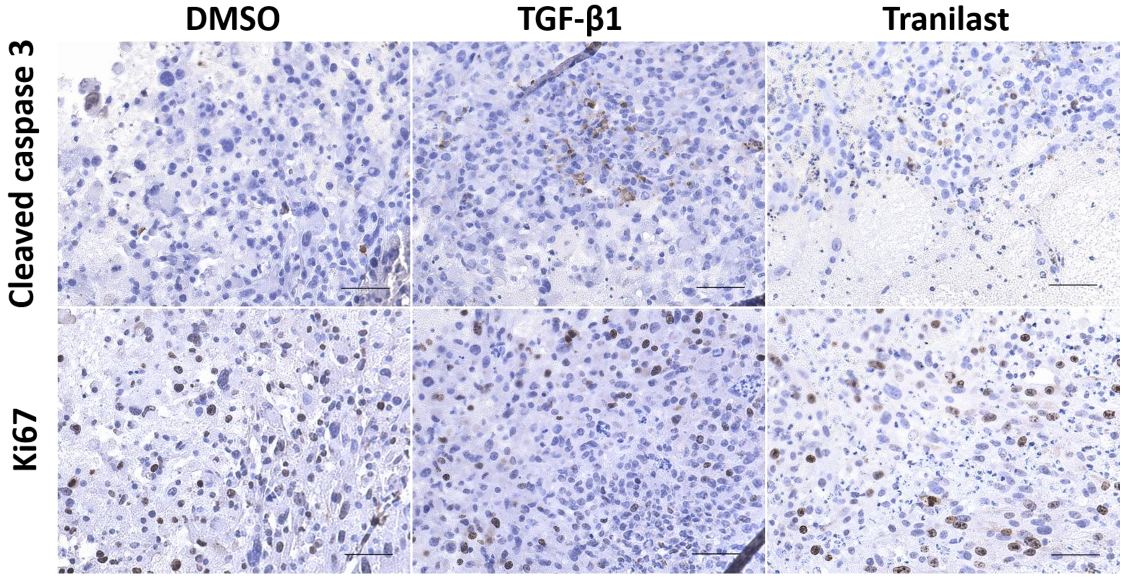

Fig. S3. Effects of tranilast and TGF-β1 on apoptosis and cell proliferation in tumor-bearing explants. Explants from Figure 5C were subjected to immunohistochemical staining for cleaved caspase 3 and Ki67 on day 4. Scale bars, 50 µm.

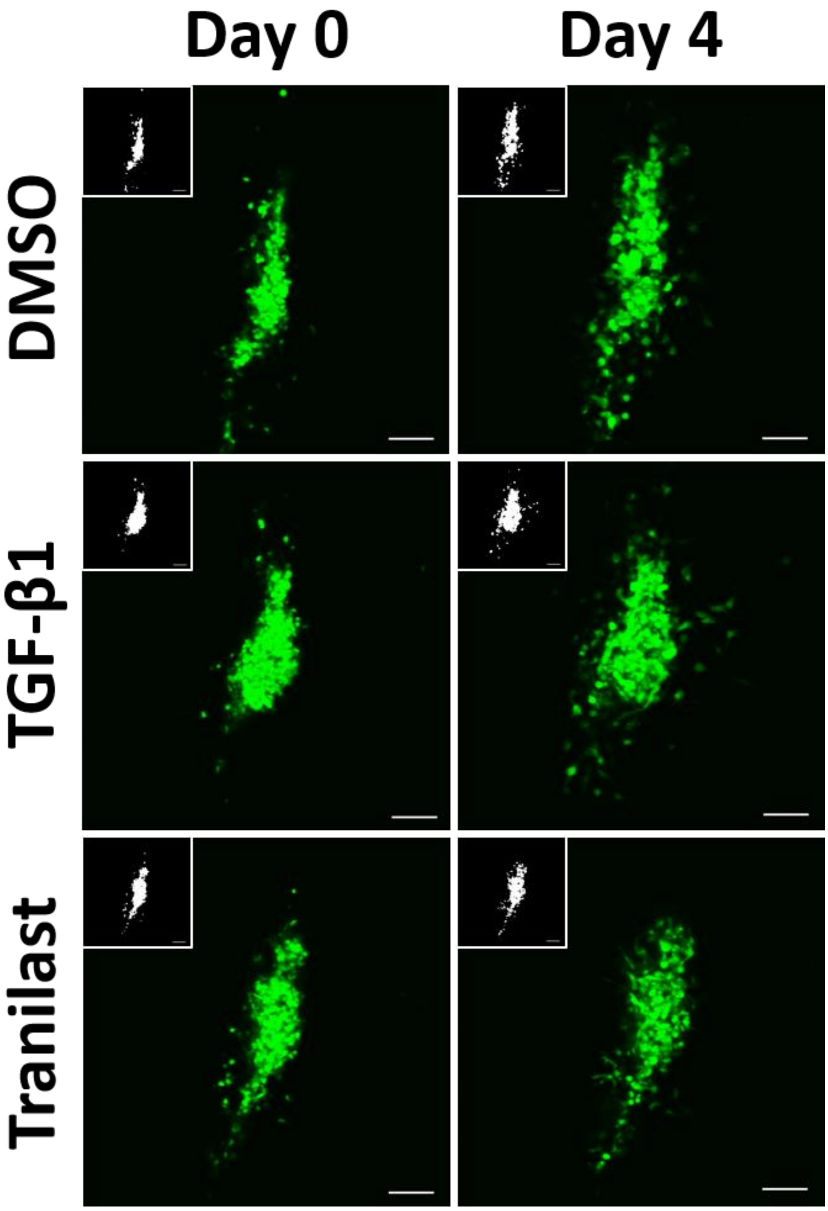

Fig. S4. Visualization of MADM mouse–derived GFP-positive *Tp53–/–;Nf1–/–* glioma cells in organotypic slices prepared from recipient C57BL/6J mice at 13 days after implantation. Slices were treated with DMSO, TGF-β1 (10 ng/ml), or tranilast (100 μM) for 0 or 4 days. Scale bars, 100 μm.

Video S1. Time-lapse imaging of MADM mouse–derived GFP-positive *Tp53–/–;Nf1–/–* glioma cells in organotypic slices prepared from recipient C57BL/6 mice. Slices were treated with DMSO, TGF-β1 (10 ng/ml), or tranilast (100 μM). Images were aquired every 30 min.
